# Children's eye health programmes: Successful strategies and challenges

**Published:** 2017

**Authors:** Asha Latha Mettla, Srinivas Marmamula, Rohit C Khanna

**Affiliations:** Associate Director, Gullapalli Pratibha Rao International Centre For Advancement of Rural Eye Care, LVPEI, Hyderabad, India; Senior Leader, Primary Health Care, Community Eye Health Education and Research, Gullapalli Pratibha Rao International Centre For Advancement of Rural Eye Care, LVPEI, Hyderabad, India; Director, Gullapalli Pratibha Rao International Centre For Advancement of Rural Eye Care (GPR ICARE), LVPEI, Hyderabad, India

**Figure:**
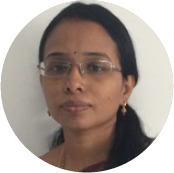
Asha Latha Mettla

**Figure:**
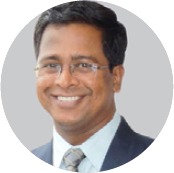
Srinivas Marmamula

**Figure:**
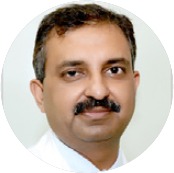
Rohit C Khanna

**School eye health programmes provide a unique opportunity to positively influence the health of 700 million children globally. The impact of school eye health (SEH) goes far beyond good vision—it encompasses education, social development and economic productivity.**

**Figure:**
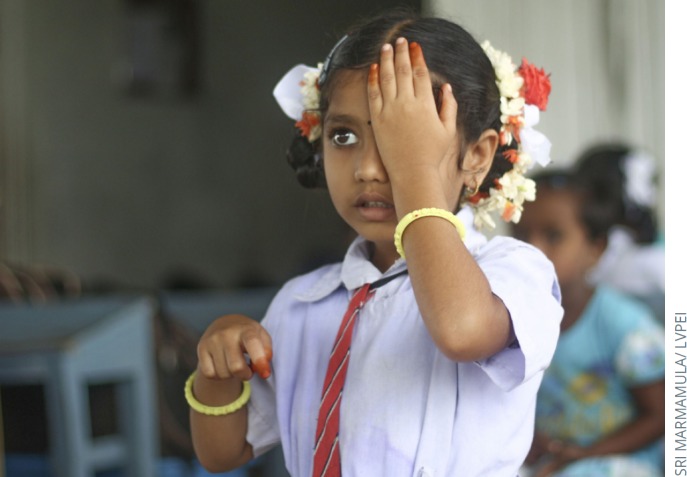
A little girl being screened at her school. INDIA

Vision plays an important role in the learning and development of a child. Globally, 19 million children, are estimated to be visually impaired.^1^ A comprehensive eye health programme should include screening in schools, *anganwadis* (pre-school), use of a key informant approach and household screening to aid in screening and identification of children with visual impairment.^2–4^ One such comprehensive eye health programme is being implemented by L V Prasad Eye Institute in India.

This programme aims to provide eye screening to all children between the ages 0 and 15 in project service areas and facilitate necessary interventions. The schematic of the programme is shown in Figure 1. School vision screening is one of the major components. In this article, key steps, strategies and challenges encountered in a school screening programme in one administrative block of Krishna District in the Indian state of Andhra Pradesh are described.

## The process

### Step 1: Obtaining necessary approvals

Necessary approvals were obtained from appropriate local government authorities such as the district collector, district education officer and key stakeholders such as *mandal* (sub-district) development officer, mandal education officer, *anganwadi* supervisor, village *sarpanch* (Head) in case of household screening in the community.

### Step 2: Teacher training

After all the due permissions were obtained, school authorities were requested to nominate one teacher from each school. All the nominated teachers were invited for one day of training at a central location. The central location was usually the *mandal* (sub-district) education office or a school. Typically, 25–28 teachers were trained at each session. The content, duration, training materials and delivery methods were standardised and provided by an experienced vision technician. This content included a brief description on the structures and functions of the eye, refractive errors, common eye conditions in children and vision screening procedures to diagnose and correct them.

Our initial research revealed a large variability in diagnostic accuracy among trained teachers compared to Community Eye Health Workers (CEHW).^5^ Although having trained teachers will add sustainability to the programmes, attributes required for a teacher to be a consistent screener remain elusive.

For further screening, CEHWs were also trained as there was variability in screening skills of teachers. An optometrist trained in detecting low vision and ophthalmologist were involved in screening children in special schools and schools for the blind.

Teachers' vision assessment was part of the teachers training programme. Of the 738 teachers screened, 400 (54.2%) were using glasses and 35 (4.7%) were referred for further examination. This assessment helped us in identifying those requiring intervention and who can potentially become an advocate for eye care for children in their respective school as well as in the community.

### Step 3: School vision screening

After training, the school authorities were approached for screening. It is sometimes difficult to get access to screen children and to examine referred children. Children who did not attend referral services, were examined by the vision technician at the schools. It took a lot of time and effort to follow up with the school authorities at the school level. Development of a tentative School Eye Health Calendar involving school authorities helped.

In the project area, there is one integrated school for the blind and three schools for intellectually challenged children. Teachers in these schools also helped in managing children while screening. An optometrist and an ophthalmologist trained in paediatric eye care conducted eye examinations and referred to the tertiary centre for further management. Referral uptake from the school for the blind was a challenge inspite of repeated visits. Service uptake from special schools was relatively better.

**Figure 1:**
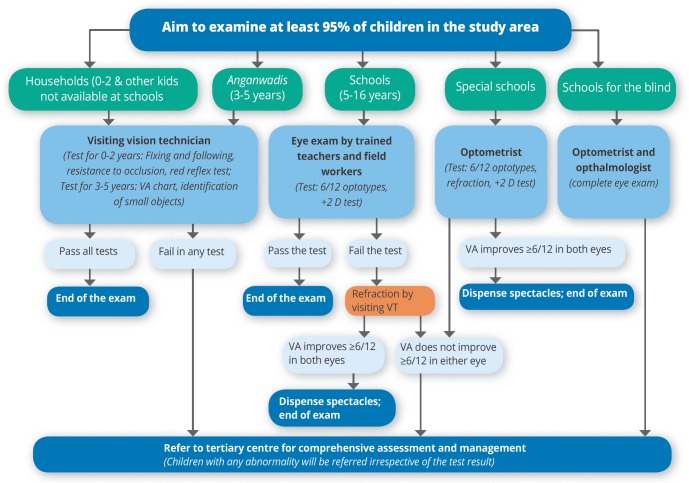
Screening, referral and management process.

### Step 4: Referral tracking

All the children who either failed the screening test or had any abnormality were referred to a primary eye care centre (vision centre, VC) in the vicinity of the school. A full time CEHW along with coordinator was assigned for referral follow up. With this system in place, the referral conversion rate was 61%. To improve upon the referral conversion rate, vision technicians went to schools and examined the children including non-cycloplegic refraction, resulting in increased uptake to 90%. Parent meetings were organised at schools for increasing awareness and uptake of spectacles. For the children, whose parents did not turn up for the meeting, teachers took the responsibility to get spectacles. Those who needed further management were referred to secondary or tertiary centres (TC). Among those referred, 41% utilised the services. However, active intervention in the form of telephone calls to parents and provision of travel cost for those who cannot afford it, improved the uptake to 61%. Considerable time and effort was required to counsel and convince parents and care givers to bring the children to access services.

In schools, where there were more than ten referrals to tertiary centres, the ophthalmologist visited the centre and examined the children. However, as this is not sustainable, a tele-consultation programme is being introduced in schools, thus avoiding travel of the child to tertiary centres.

### Step 5: Key informants

As part of the programme, key informants such as *Anganwadi* teachers and ASHA (Accredited Social Health Activist) workers were asked to inform the CEHW if they suspected any child with visual impairment. The children identified were referred to the vision technician.

## Management Information System for school screening

This was developed to track and report the number of children referred and attended. A framework for monitoring the school vision screening programme included:

- Developing and assigning a unique ID system to identify each child in the project.
- Process indicators were developed, which included average screening per CEHW, primary referral conversion rates (conversion at vision centre), secondary referral conversion rate (conversion at secondary and/or tertiary centres) and spectacle uptake on a monthly basis.
- Output indicators included were number of schools covered, children screened and children referred.
- Data collection forms populated by school with a summary report highlighted the response rate.
- Referral register for recording details of children who failed screening tests by CEHW highlighted the service provided.
- Referral conversion details were tracked weekly
- Structured weekly reporting formats, describing the weekly activity and plan for the next week were developed.

**Figure 2:**
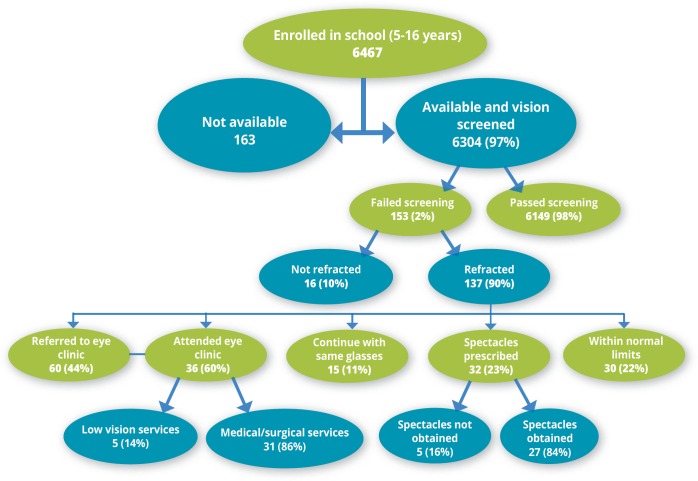
Coverage and service uptake in one block of Krishna District, Andhra Pradesh.

Monitoring and evaluation helped us ensure the quality of screening and review of service uptake. As of date, 2,43,695 children were screened in sub-districts, out of which 11,412 (4.7%) were referred. Among these, 9,546 (84%), children attended the vision centre. Follow up services are ongoing. Data flow of a sub-district is presented in Figure 2.

One of the major issues identified with this programme was the poor uptake of services and additional intervention required to increase the referral uptake. Additional intervention may be an important aspect that has to be considered if the programme has to be replicated sustainably on a large scale.

## Barriers

Children who did not turn up for services at the vision centre level and TC level were identified. Parents were contacted by the CEHW. Responses were collected from 61 parents whose children did not attend vision centre and from 22 parents who did not attend a TC. Major barriers identified are lack of time (22%), lack of priority to eye health (18%) and no one to accompany the child (10%).

## Way forward

If the programme has to be scaled up and be sustainable, it is essential to identify the right teachers who can help with the initial screening. At the same time, there is a need for a qualified technical team, including a technician and an ophthalmologist, to ensure that the referrals are examined. Awareness of parents and school teachers is also a priority. Once, spectacles are delivered, systems need to be put in place to ensure compliance with spectacles usage. It is also essential to measure the impact of the intervention in terms of change in scholastic performance. Use of technology for data collection like tablet-based applications and cloud storage may help for rapid data collection and real-time monitoring. This could be an important factor for replication to a large scale or to support a nationwide programmes.

## Acknowledgements

This project is supported by United States Agency for International Development (USAID), Lions Clubs International Foundation, Sun Pharma and Hyderabad Eye Institute.
